# Oral nutritional supplement combined with an online resistance training programme to improve physical function and nutritional status in older adults receiving home care and at risk of sarcopenia:  protocol for the randomised controlled POWER trial

**DOI:** 10.12688/hrbopenres.14086.3

**Published:** 2026-01-20

**Authors:** Catherine M. Fallon, Caitriona G. Cunningham, Katy Horner, Clare A. Corish

**Affiliations:** 1 UCD School of Public Health, Physiotherapy and Sports Science, University College Dublin, Belfield, Dublin 4, Ireland; 2 UCD Institute of Food and Health, University College Dublin, Belfield, Dublin 4, Ireland

**Keywords:** older adults, home care, sarcopenia, oral nutritional supplement, online resistance training

## Abstract

**Background:**

Risk of sarcopenia and poor nutritional status are observed in older adults receiving home care. Hence, the aim of the POWER trial is to investigate the effectiveness of a whey protein oral nutritional supplement (ONS) combined with an online resistance training (RT) programme compared to RT alone on physical function, nutritional status and health-related outcomes in community-dwelling older adults receiving supportive home care who are at risk of sarcopenia.

**Methods:**

This home-based, randomised controlled trial will include older adults aged ≥70 years, receiving supportive home care (professional and/or informal), who will be screened for sarcopenia via telephone. Forty-six participants will be randomised into either (i) ONS + RT or (ii) RT only trial arms. Participants in the ONS + RT group will be provided with a whey protein ONS enriched with leucine and vitamin D to consume day daily for 12 weeks. All participants will be provided with an online RT programme twice weekly via Zoom. The primary outcomes are physical function, measured using the Timed Up and Go test and nutritional status, measured using the Mini-Nutritional Assessment-Full Form. Secondary outcomes include body composition, dietary intake, gait speed, muscle strength, cognitive function, depression risk, activities of daily living, quality of life and feasibility of intervention implementation. All outcomes will be measured at baseline (T1), post-intervention (T2) and 12 weeks post-intervention (T3).

**Conclusion:**

This study will provide data on the effectiveness of a whey protein ONS enriched with leucine and vitamin D combined with an online RT programme delivered via Zoom, compared to the RT programme alone, for older adults at risk of sarcopenia and receiving supportive home care. If effective, this combined approach could act as an important strategy to support maintenance of physical function and nutritional status in older adults, for whom home-based interventions are likely a more feasible option.

**Trial registration:**

NCT05688956; registered December 2022.

## Background

Muscle loss is caused by a discrepancy between muscle protein synthesis and muscle protein breakdown ^1^, which can be contributed to by ageing, inadequate nutrition and physical inactivity ^2, 3^. Sarcopenia is an age-related condition defined by the European Working Group on Sarcopenia for Older People (EWGSOP) as low muscle strength, mass and physical function ^1^. This can lead to reduced ability to carry out activities of daily living (ADLs), falls, loss of independence, hospitalisation, disability and death ^4, 5^. When physical function significantly deteriorates, supportive formal or informal home care to perform ADLs may be required ^6, 7^.

Currently, the reported prevalence of sarcopenia among community-dwelling older adults (≥60 years) ranges from 5–10% ^8– 10^. Within residential care homes, reported sarcopenia prevalence is between 17–73%, and from 22–87% within assisted living facilities ^11^. Older adults’ response to protein ^12– 14^ and resistance training (RT) ^2, 15^ is blunted compared to the response of healthy younger populations. With reduced mobility, anabolic resistance is further exacerbated in older adults ^13, 16^. The relationship between sarcopenia and undernutrition is complex, with the development of one interlinked with the other ^2, 5^. Undernutrition, mainly when there is insufficient energy and/or protein, contributes to muscle protein breakdown ^3, 5^. Older community-dwelling adults receiving home care are more frequently undernourished [14.9%, 95% CI: 9.9–20% compared to those not requiring home care 4.7%, 95% CI: 3.6–6.1%] ^17^.

To increase muscle strength and mass, nutritional interventions and/or RT are recommended ^18, 19^. The functional benefits of taking part in RT include improved mobility, performance in ADLs, resistance to injuries and falls and enhanced overall quality of life in older adults ^20– 23,^. Mobility limitations can persist in individuals who are at a higher risk of lower protein intake (<0.8 g/kg adjusted bodyweight/day) ^24^. Older females, with higher BMI and poor appetite are at higher risk of inadequate protein intakes ^25^. Increasing energy ^26, 27^ and protein (1–1.5 g/kgBW/day) ^26– 28^ intakes to prevent malnutrition and muscle loss is recommended for older adults ^26– 28^. To further stimulate muscle protein synthesis, increasing leucine intake by up to 2.8–3 g twice/day is recommended ^29, 30^. Older adults residing in the Northern Hemisphere should also take a daily vitamin D supplement (15 µg for healthy older adults and 20 µg for home bound older adults) ^19, 31– 33^. Providing ONS fortified with whey protein, leucine and vitamin D has shown promise in improving health-related outcomes ^34, 35^ while such ONS combined with RT in older adults with sarcopenia have shown positive effects on muscle strength, body composition and physical function ^34^.

Older adults with sarcopenia, undergoing physical rehabilitation, demonstrated improvements in physical function [Timed Up & Go (TUG) and gait speed) and nutritional status (Mini-Nutritional Assessment-Full Form (MNA-FF)] following 8 weeks of ONS enriched with leucine and vitamin D and supervised RT compared to an isocaloric placebo supplementation and supervised RT ^36^. These findings demonstrate the added benefits of nutritional supplementation to improve outcomes of an exercise programme in hospitalised older adults. However, whether similar effects are observed in older adults receiving supportive home care is unknown. Older adults with sarcopenia are recommended to perform at least two RT sessions per week ^18, 37^. Given that a number of studies indicate older adults are less likely to engage in exercise programmes for varying reasons ^38, 39^, supervised exercise programmes delivered via an online platform are worth considering ^40, 41^. Online platforms such as Zoom provide a potential and accessible solution for older adults who have mobility and/or transportation limitations ^41– 43^. Providing a supervised and consistent programme can improve adherence ^44^ plus the group-based aspect can provide a social element ^41^. Recent studies indicate overall acceptability of online exercise programmes among healthy older adults ^41, 44^.

Hence, the aim of this study is to test the effectiveness of a new whey protein ONS enriched with leucine and vitamin D (Fortimel Advanced, Nutricia) combined with an online RT programme versus the RT programme alone on the physical function and nutritional status of older adults receiving supportive home care who are at risk of sarcopenia. The primary outcomes of the study are 1) physical function as measured by the TUG and 2) nutritional status as measured by the MNA-FF.

Trial registration: NCT05688956; registered December 2022.

## Methods

### Study design

**Figure 1.**
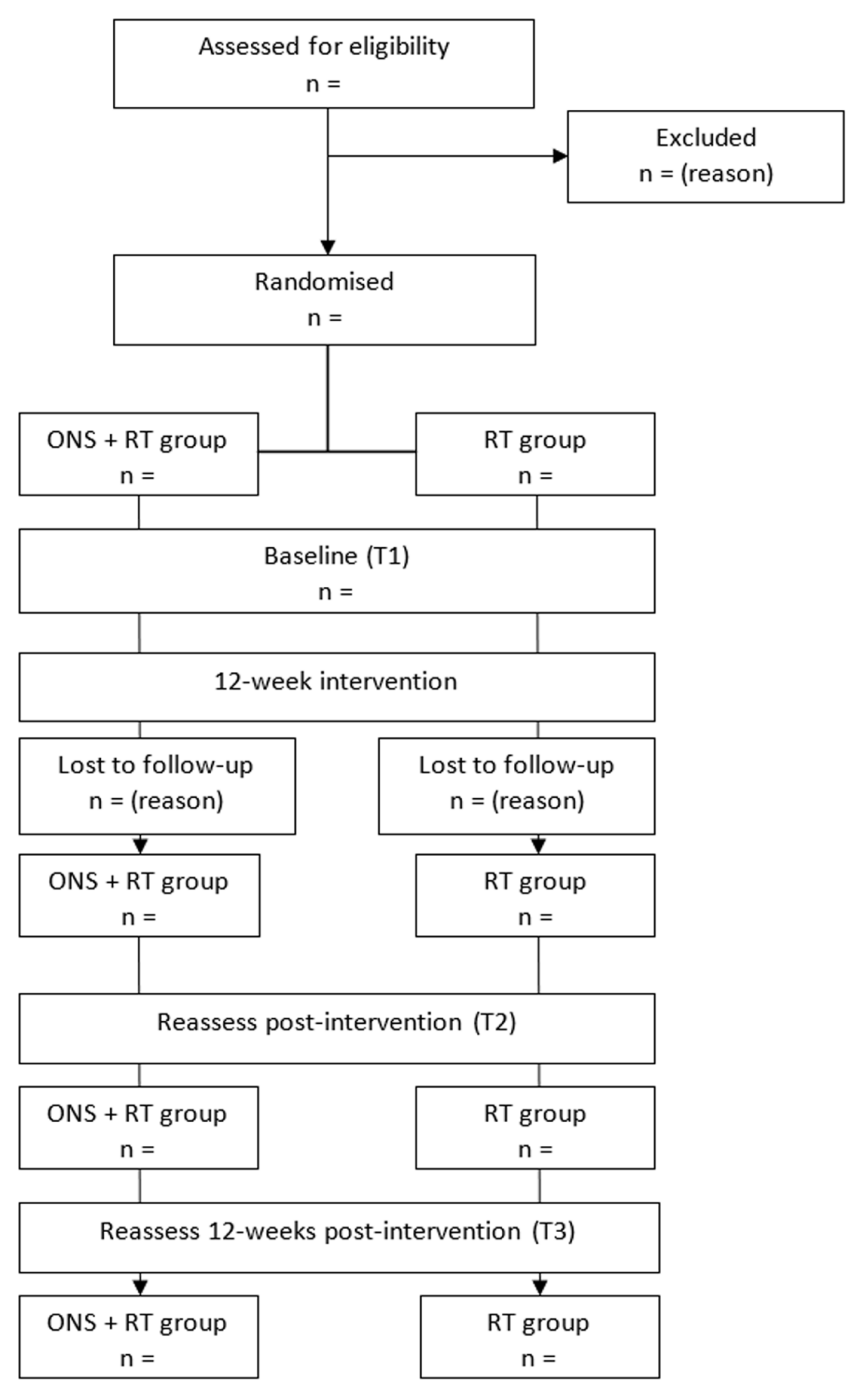
Study design and participant flow.

This randomised, parallel group, 12-week clinical trial will include 46 community-dwelling (residing in either an urban or rural setting in their own home) older adults who receive supportive home care and are at risk of sarcopenia. Participants will be randomly allocated to either the (i) ONS + RT or (ii) RT only trial arm. Screening potential participants will be undertaken via telephone. Baseline (T1) and follow-up (T2 & T3) assessments will be undertaken in the participant’s own home. Post-intervention (T2) testing will be conducted immediately after the final RT session and again 12 weeks later (T3). The study design and participant flow chart are shown in Figure 1 ^45^.

### Sample size

Study sample size calculation was conducted in G* Power (version 3.1). Based on previous study findings ^36^, 15 participants per group are required to detect a mean difference of 3.7 seconds in time to complete TUG with 80% power and significance at p<0.05. Additionally, based on the same findings ^36^, 16 participants per group will be needed to detect a mean difference of 1.52 in MNA-FF score between the intervention group and the control group with 80% power and significance at p<0.05. To ensure an adequate sample size to enable per protocol analysis, the sample size was increased to 23 participants per group ^46^. This will allow for both intention-to-treat and per-protocol analyses, ensuring rigorous analysis of intervention effectiveness.

### Eligibility criteria, recruitment and screening

Older adults who are ≥70 years old, community-dwelling and receiving regular supportive home care from a professional and/or informal carer ^47^ will be invited to take part in the research study. A variety of recruitment techniques will be employed including contacting home care associations, media coverage, poster distributions and face-to-face strategies such as presenting at community centres, day centres and exhibition shows for older adults. Non-profit organisations and charities that support older adults will also be contacted and asked to share information about the study. The inclusion and exclusion criteria are summarised in Table 1. Individuals will be screened for eligibility using two screening questionnaires conducted via telephone: i) SARC-F questionnaire with a cut-off score of ≥1 (out of 10) to screen for risk of sarcopenia ^48^ ( Table 1) and ii) the Physical Activity for Readiness Questionnaire for Everyone (PARQ+) to assess participants’ readiness to participate in exercise. Older adults with moderate to severe cognitive (MMSE <24) impairment will be excluded due to the need for older adults to follow instructions independently at home to deliver an effective exercise dose and our concern about potential risk of injury given the online delivery of the resistance training programme.

**Table 1.** Inclusion and exclusion criteria for the POWER Study.

Inclusion Criteria	Exclusion Criteria
1. Older adults (70+ years) 2. Community-dwelling 3. Receiving supportive home care (professional and/or informal) 4. At risk of sarcopenia (SARC-F ≥1) ^48^	1. Cognitive impairment (MMSE <24) ^53^ 2. Severe kidney disease (glomerular filtration rate <30 mL/min) 3. Moderate to severe liver disease (Child-Pugh class B or C) 4. Psychiatric disorder 5. Receiving active treatment or palliative care for cancer 6. Receiving enteral or parenteral nutrition 7. Allergic to dairy products 8. Known hypersensitivity to any component of the ONS 9. Currently consuming ONS 10. Regularly undertaking resistance training 11. Advised by GP not to undertake exercise

### Randomisation and blinding

On meeting the eligibility criteria, participants will be randomised into either the (i) ONS + RT or (ii) RT only, using a 1:1 ratio by an independent researcher not involved in the POWER study using the Clinical Trial Randomization Tool hosted by the National Cancer Institute. Eligible participants will be assigned an identification number and then added to the study database. It will not be possible to blind the participant or researchers to the intervention allocation.

### Interventions

***Oral Nutritional Supplements***

Participants randomised to ONS + RT will consume two 200 mL bottles of whey-based protein ONS enriched with the amino acid leucine and vitamin D each day ^49^. On exercise days, the first ONS will be consumed at the time of day when the least amount of protein is normally consumed and the second ONS will be consumed as soon as possible after the RT session. On non-exercise days, the most appropriate time for consumption to optimise potential muscle protein synthesis will be based on a 24-hour dietary recall, undertaken at the baseline assessment ^50^. The nutritional content of the ONS (Fortimel Advanced, Nutricia) per 200 mL includes: energy: 300 kcal; protein: 20.8 g; leucine: 3 g; fat: 10.4 g; carbohydrate: 30 g; dietary fibre: 2.8 g; vitamin D: 10 µg (Appendix B) ^45^.

***Dietary advice***

All participants and home support person, if required, will be provided with written dietary information (“ Making the Most of Every Bite”, a high energy and protein cookbook, available from the Irish Health Service Executive). Additional dietary advice will verbally be provided to describe protein sources, portion sizes, timings and distribution of protein intake.

***Online resistance training programme***

A 12-week, progressive, group RT programme, developed with a registered physiotherapist and an exercise physiologist, will be delivered to all participants in their own home via Zoom twice per week by a health science researcher with exercise training (Exercise Instructor). Each RT session will last approximately 45–60 minutes, will include a warm-up and cool-down and moderate to high intensity RT exercises rated on the 10 point Borg Rating of Perceived Exertion (RPE) scale ^51^. During the baseline assessment, the exercises will be demonstrated, and the appropriate RT load will be determined using the 5- Repetition Maximum method ^52^.

The RT programme will include upper and lower body strengthening exercises, which can be modified to each participant’s muscle function, aiming for 2–3 sets of 8–12 repetitions. Exercises include sit-to-stand, shoulder press, bicep curl, calf raise, leg extension and lateral arm raise. Throughout the sessions, the exercise conducted, weight lifted during the exercise, sets and repetitions performed and RPE will be recorded ( Table 2). Following the T2 post-intervention assessment, participants will not receive the online RT programme. At the T2 assessment, they will be encouraged to continue to exercise but no additional supervised sessions or support will be provided between T2 and T3.

**Table 2.** Progression plan from week 1 to 12 for the resistance training programme (2 sessions/week).

Phase	Week	Intensity	Duration	Repetitions	Sets	Exercise
Phase 1	Week 1–2	Easy-moderate	45 minutes *	8–10	2–3	Sit-to-stand, Shoulder press, Bicep curl, Calf raise
Phase 2	Week 3–5	Easy-hard	45–50 minutes *	10–12	3	Sit-to-stand, Shoulder press, Bicep curl, Calf raise
Phase 3	Week 6–8	Moderate-hard	60 minutes *	12	3	Sit-to-stand, Shoulder press, Bicep curl, Calf raise, Leg extension, Lateral arm raise
Phase 4	Week 9–12	Moderate-hard	60 minutes *	12	3	Sit-to-stand, Shoulder press, Bicep curl, Calf raise, Leg extension, Lateral arm raise

*Rest period is the same throughout the whole programme: 1–2 minutes between sets; 2–3 minutes between exercises and at least 48 hours between sessions

### Data collection

Initial screening data will be collected over the telephone, which will capture home care status, SARC-F score, MNA-short form score, and demographic data. Written informed consent will be obtained at the baseline assessment in the participant’s home (Appendix C) ^45^. Outcome data will be collected at baseline (T1), post-intervention (T2) and 12-weeks post-intervention (T3) in the participant’s home and will be recorded on an online form (Google Forms). If required, assistance with data collection will be provided by the participant’s home carer. Adherence to the ONS will be recorded post-intervention. Adherence to the RT programme will be monitored and recorded throughout the study intervention (T2) ( Table 3).

**Table 3.** Measures at the three study timepoints.

TIMEPOINT	T1 Baseline	T2 Post-intervention	T3 12 weeks post- intervention
ENROLMENT			
Eligibility	☒		
Randomisation	☒		
Written informed consent	☒		
OUTCOMES			
Timed Up & Go	☒	☒	☒
Mini Nutritional Assessment-Full Form	☒	☒	☒
Body composition	☒	☒	☒
Dietary recall	☒	☒	☒
4-metre gait speed	☒	☒	☒
Handgrip strength	☒	☒	☒
Five times Sit-To-Stand	☒	☒	☒
Cognitive function	☒	☒	☒
Depression risk	☒	☒	☒
Activities of daily living	☒	☒	☒
Quality of life	☒	☒	☒
Number of participants recruited	☒		
Number of participants retained		☒	☒
Adherence to oral nutritional supplement		☒	
Adherence to resistance training		☒	
Acceptability of oral nutritional supplement		☒	
Acceptability of resistance training		☒	

### Study outcomes

***Primary outcomes***

The primary study outcomes are physical function, assessed using TUG ^54^ and nutritional status, determined using the MNA-FF ^55^.

**Timed Up and Go**

Participants will be timed when asked to stand up from a standardised armchair (46 cm seat height), walk 3 metres, turn around, walk back and sit down ^54^. The use of a walking aid will be permitted. The following reference values will be used: 9.2 (8.2–10.2) seconds for 70 to 79 years, and 11.3 (10–12.7) seconds for 80 to 99 years ^56^. A cut-off time of 20 seconds ^1^ or those unable to complete the TUG due to health problems will be considered low physical function ^57^. The TUG has demonstrated good inter-rater and intra-rater (intraclass correlation coefficient = 0.99) reliability ^54^.

**Mini-Nutritional Assessment-Full Form**

The MNA-FF will be used to assess the nutritional status of participants. The MNA-FF includes 18 questions to assess an individual’s nutritional status ^55^. The MNA-FF includes anthropometric measures and questions on general health status, dietary habits and self-perceived health and nutrition status ^58^. A score of 24–30 indicates that an individual is normally nourished, 17–23.5 that an individual is at risk for malnutrition, and a score of <17 that the person is malnourished ^55^. The MNA-FF has demonstrated high sensitivity (96%), specificity (98%), and predictive value (97%) in accurately identifying the nutritional status of older adults compared to clinical assessment ^59^.

***Secondary outcomes***

**Body composition**

Height will be measured with a stadiometer, with shoes removed. In participants who are unable to stand, height will be estimated by measuring ulna (forearm) length (cm) with reference to predictive equations ^60^. For ulna length, the distance between the olecranon process and the midpoint of the prominent bone of the wrist (styloid process) will be measured with the left arm folded across the chest with the fingers pointing towards the shoulder ^60, 61^.

Participants’ body composition [fat (kg/%), muscle (kg/%), and weight (kg) and BMI (kg/m ^2^)] will be assessed using the Tanita MC-780MA (2015, Tanita Corporation, Japan), an 8 electrode multi-frequency segmental body composition analyser ^62, 63^.

Calf and mid-upper arm (MUAC) circumferences will be measured. Calf circumference will be measured with a non-elastic measuring tape using the widest part of the calf ^64^. MUAC will be measured using the same tape, at the midpoint between the acromial process of the shoulder and the olecranon process of the elbow with the arm hanging relaxed at the participant’s side ^65^. Measurements will be taken while seated.

**Dietary intake**

A 24-hour dietary recall using a multiple pass technique will be conducted at each home-based assessment to establish the dietary pattern of each participant ^66^. Participants will be asked to recall all foods and drinks consumed in the previous 24 hours, commencing with first intake of food or drink of the day. Any dietary supplement(s) consumed will also be recorded. The list of foods will be repeated back to the participant, and using closed questions, each participant will be asked about foods frequently omitted (e.g., sauces, dressings, beverages, sweeteners, etc.), timing of meals and additional snacks. A detailed description of food consumed (type, brand, weight) will be recorded. The dietary intake data will be reviewed for a third time with the participant to validate the information. The 24-hour dietary recall data will be analysed using Nutritics ^TM^ software (version 5.96). Intakes of protein (g) and energy (kilocalorie) will be calculated as g/kgBW and as kilocalorie/kgBW respectively. Changes in macro and micronutrient intakes between all three timepoints will be assessed.

**Gait speed**

Gait speed will be measured using 4-metre (m) gait speed. A 4-m flat and unobstructed course will be marked out by tape. Participants will be asked to walk at their usual speed. Once the participant’s foot is across the finish line, the time taken to complete the course will be recorded ^67, 68^.

**Muscle strength**

Handgrip strength will be measured as an indicator of upper limb muscle strength using a dynamometer (Jamar hydraulic hand dynamometer) ^69^. Participants will be asked to squeeze the dynamometer handle with force, with the arm at a 90-degree angle in the seated position. Each participant will complete the test three times with each hand to get an average ^69^. The 2019 EWGSOP cut-offs to define low handgrip strength (<27 kg for males and <16 kg for females) will be used ^1^.

The Five times Sit-To-Stand (5xSTS) will be used to assess lower limb muscle strength ^70^. Participants will be asked to stand from the chair five times as quickly as possible and the time taken to complete the assessment will be recorded ^70^. Using the 2019 EWGSOP cut-off, low muscle strength will be defined as >15 seconds ^1^.

**Cognitive function, depression risk and activities of daily living**

Cognitive function will be measured with the Mini-Mental State Examination (MMSE) ^49^. Cognitive function impairment is defined as a score <24 points and such individuals will be excluded from the study ^71^. The Geriatric Depression Scale (GDS-15) will be used to screen for depression risk ^72^. This scale assigns scores of zero or one to each response, with a score >5 indicates risk of depression ^72^. The ability of participants to perform ADLs will be assessed using the Katz Index of Independence in ADLs ^73^. This Index assesses six functional activities: bathing, dressing, toileting, transferring, continence and feeding ^73^.

**Quality of life**

Quality of life will be assessed using the Short Form (SF)-12 × 2 ^®^^74^. This instrument uses twelve questions to assess perceived general health (physical and social function, pain, emotion and mental health) ^74^. The physical component score indicates physical health status and the mental component score indicates psychological well-being ^74^.

**Feasibility of intervention implementation**

Recruitment and Retention

Recruitment rate will be defined as the percentage of eligible participants who consent to participate in the research study (total number of participants recruited divided by the total number of people screened for eligibility multiplied by 100). Retention rate will be defined as the percentage of participants who start and go on to complete the study intervention. The number of participants who complete the follow-up assessment (T3) will also be recorded. Reasons for study dropout will be recorded.

Adherence to oral nutritional supplement

Participants will be asked to self-report intake of ONS per day using a paper copy ONS recording sheet that provides instructions on how to consume the ONS. Participants will check two tick boxes to indicate they have consumed two ONS on the day of consumption and tick one box if only one ONS is consumed. If they have not completely consumed a bottle, they will be asked to record this for the specific day. Participants will be asked to retain both used and unused ONS bottles; these will be counted at the end of the intervention. Adequate adherence will be defined as consuming an average of 10 out of 14 (71%) ONS per week over the 12-week intervention ^75^. Reasons for non-adherence will be recorded.

Adherence to resistance training

Adherence during the supervised RT session will be monitored by the Exercise Instructor who will record attendance, number of repetitions, sets, weight used, and the RPE on the study online recording database. Adequate adherence will be defined as successfully participating in 18 out of 24 sessions (>75%) ^76^. Reasons for non-adherence will be recorded.

Acceptability of the oral nutritional supplement

Acceptability of the ONS will be assessed post-intervention with three questions using a 9-point Hedonic scale to evaluate flavour, texture, and overall acceptance ^77^. Additionally, four questions will utilise a 5-point Hedonic scale to assess visual appearance, smell, taste, and aftertaste of the ONS ^77^. Participants will also respond to a final question regarding their willingness to purchase the protein drink, with options ranging from "would certainly buy" to "would certainly not buy" ^77^.

Acceptability of the resistance training programme

A short bespoke questionnaire completed by participants post-intervention will be used to evaluate their perceptions of the RT programme. All questionnaire statements are framed positively, and participants will be asked to rate their agreement with each statement on a five-point Likert scale ranging from “Strongly disagree” to “Strongly agree”. Participants’ responses will be collated and summarised ^78^.

### Adverse events

Should an adverse event occur, it will be discussed immediately with the research advisory team (CC, CCu, KH) and participants will be advised to contact their general practitioner as appropriate. As per ethical approval, the occurrence of any unexpected serious adverse event will be reported to the UCD HREC ( hrec@ucd.ie).

### Data management

Personal data will be stored in a password protected university database in compliance with the Data Protection Act 2018 ^79^. Only the researchers will have access to this information. Each participant will be assigned a study identification number, which will be used to pseudonymise the data collected. All data will be pseudonymised for analysis, enabling the potential re-linking of individual participant data for data checking if necessary. All data will be anonymised after four years. No personal data will be transferred to Nutricia Ireland Ltd as per the signed legal agreement between UCD and Nutricia Ireland. Study data will be made available to researchers upon reasonable request.

### Statistical analysis plan

Statistical analyses will be performed using SPSS Statistics for Windows, version 27 (SPSS Inc., Chicago, Ill., USA) and RStudio (2023.06.2. Posit, PBC) as appropriate. Data cleaning will be conducted prior to analysis.

Demographic data and study primary and secondary outcomes will be described using measures such as mean and standard deviation, median and interquartile range, 95% confidence interval, or count and percentage. Descriptive statistics will also be used to report the feasibility of the intervention. Missing data will not be imputed for the primary analysis.

Data will be analysed using the intention-to-treat approach, wherein all participants who have been randomly allocated to the study intervention ^80^ will be included. The between-group and within-group difference at baseline (T1), post-intervention (T2) and 12-weeks post-intervention (T3) in primary and secondary outcomes will be analysed using either parametric or non-parametric, based on the normality of data distribution. The primary outcome is TUG and MNA-FF post-intervention (T2). Further, a mixed between-within linear model will be used to assess group, time, and group x time interaction effects. Between-group linear mixed-model analysis will be used to assess the mean difference in change from baseline to post-intervention. The dependent variables will be TUG and MNA-FF with group (ONS + RT, RT), time (baseline, post-intervention), and the group-by-time interaction as fixed factors; participant ID as a random factor; and baseline values as covariates. Effect sizes (partial eta squared) will be calculated using the Cohen’s d classification (0.01 = small, 0.06 = moderate, 0.14 = large). Variables not normally distributed will be transformed as appropriate. The proportion of participants whose TUG and/or MNA-FF improved or deteriorated post-intervention (T2) will be calculated. The interindividual variability in response to the ONS will be calculated using the Atkinson and Batterham formula ^81^.

Using the same approach as above, between-group differences and within-group differences at baseline (T1), post-intervention (T2), and 12 weeks post-intervention (T3) will be analysed for secondary outcomes.

A per-protocol analysis will also be conducted, including participants who adhered to the intervention and have baseline (T1) and post-intervention (T2) measurements ^82^.

A p-value of less than 0.05 will be considered statistically significant.

### Qualitative data analysis plan

The free text comments of the acceptability questionnaires will be used to further explore the acceptability of the ONS and online RT intervention and relevant barriers and facilitators to the intervention. Braun and Clarke’s framework of thematic analysis will be used to identify themes and subthemes. This includes familiarisation with the data, creating initial codes, searching for themes, reviewing potential themes and, finally, defining themes and subthemes.

### Funding

The POWER study is funded by Nutricia Ireland Ltd. The funder will have no role in the study design, conduct, data analysis, interpretation of results or write up of the study findings. A signed legal agreement between UCD and Nutricia Ireland specifies that the purpose of the grant funding is to carry out a study and the funds are to support genuine independent research, advancement of science and education, or patient and public education.

### Dissemination of findings

The study results will be disseminated through peer-reviewed journal article(s) and presented at relevant scientific conferences. The study will be disseminated to healthcare professionals such as community nurses, dietitians, gerontologists and physiotherapists as well as to volunteer organisations for older adults. The results of the study will be reported to the funder (Nutricia Ireland Ltd).

## Discussion

Interventions to improve the physical function and nutritional status of older adults with sarcopenia risk are needed ^5, 83^. The POWER Study aims to evaluate the combined effects of a novel whey protein ONS combined with an online RT programme compared to the RT programme alone on TUG and MNA-FF in community-dwelling older adults at risk of sarcopenia who receive supportive home care.

ONS are commonly prescribed in clinical settings to improve dietary energy and/or protein intake in those unable to take adequate nutrition through food sources alone ^84^. The ONS provided in this study contains whey protein, shown to stimulate muscle protein synthesis in older adults ^85^. It is also enriched with the essential amino acid leucine and vitamin D, both of which have been previously shown to have additional benefits on muscle strength, mass and physical function in older adults ^34, 85^. Two servings per day will enable participants to comply with European Society for Clinical Nutrition and Metabolism guidelines for ONS (600 kcal and 40 g protein daily) ^84^.

RT is a safe strategy to increase muscle mass, strength and physical function in older adults with sarcopenia ^86^. Providing RT online can make exercise more accessible to those who lack transport and, if supervised, can improve adherence since participants are monitored ^87^. A recent systematic review and meta-analysis concluded that RT combined with whey protein supplementation in older adults with sarcopenia improved grip strength, increased energy and protein intake and reduced inflammatory markers compared to placebo ^88^. Moreover, whey protein supplementation had a significant effect on gait speed, ADL scores, and blood biomarkers, including albumin, Insulin-like Growth Factor-1 and 25-hydroxyvitamin D ^88^. However, given that the studies conducted to date varied in type and intensity of RT, protein supplement quantity and intervention duration, the authors advised that more studies should be conducted to validate their review findings and further explore the effects of whey protein and RT in patients with sarcopenia ^88^. Furthermore, older adults receiving supportive home care are an often-overlooked cohort in multicomponent interventions who deserve consideration ^89^.

This online RT programme, developed with a registered physiotherapist and exercise physiologist, follows the American College of Sports Medicine guidelines ^90^ and sarcopenia RT prescription recommendations ^18^. This intervention will provide a structured, supervised exercise programme, encouraging participants to stay committed, which will aid adherence ^44^. Exercises will be appropriately modified and monitored throughout the intervention. As with other programmes delivered via an online platform, a limitation to this study will be that some older adults will not have access to Zoom (via laptop or tablet), therefore limiting their ability to participate. However, it provides a viable alternative for those unable to access a centre or facility for in-person RT programmes ^91^.

Assessments 12 weeks post-intervention (T3) will examine the long-term effects once the intervention ceases. Although participants may show good adherence to the online RT programme, they may discontinue RT once the programme stops ^92^. Therefore, the sustained effects of the interventions on physical function, nutritional status and health-related outcomes will be examined 12 weeks post-intervention.

A limitation of this study includes lack of a placebo supplement; therefore, intervention blinding will not be possible. A specific stipulation by the UCD Ethics Committee, given the vulnerability of the study population, was that dietary advice should be provided to all participants to promote an increase of dietary protein through food sources. Providing dietary advice to all participants may act as a confounding factor, making it challenging to isolate the effects of the ONS from changes due to the dietary advice. In addition, as both groups are undertaking the online RT programme, following a similar design to previous research in a vulnerable population ^36^, the effectiveness of the RT programme alone compared to no exercise is not possible to determine. However, in addition to the primary analysis comparing the outcomes between ONS + RT versus RT alone, the statistical approach will allow for main effects of time to be identified across both groups, which can be interpreted in relation to minimal clinically important differences. Furthermore, collecting biomarkers of muscle protein synthesis or 25-hydroxyvitamin D (25(OH)D) level will not be possible. Older adults who are housebound in Ireland are recommended a daily supplement of 20 µg of vitamin D, due to limited sun exposure and insufficient intake through food ^33^. Hence, participants currently taking vitamin D supplements will not be excluded from the current study.

## Conclusion

In summary, the POWER Study will provide data on the effectiveness and feasibility of an ONS combined with a 12-week online RT programme compared to the RT programme alone, in older adults with sarcopenia risk who receive supportive home care. It will also provide information on whether any changes observed following the intervention are maintained, 12 weeks post intervention. This will contribute to knowledge of how these interventions affect physical function and nutritional status and inform future research and practice.

## Ethics

### Declaration of Helsinki

The study will be conducted in compliance with the Helsinki declaration ^93^.

## Approvals

Ethics and consent to participate: The study was submitted for ethical approval to the UCD Human Research Ethics Committee (HREC) on 24 October 2022. Following review and addressing queries, the study was approved (LS-22-59-Corish) on 31 January 2023. The POWER study was registered on ClinicalTrials.gov (NCT05688956) in December 2022. To broaden recruitment within the Irish Health Service Executive (HSE), the study was submitted to and approved by the Clinical Research Ethics Committee Galway (C.A 3058; 6 July 2023) for Community Healthcare Organisation (CHO) area 2 (Co. Galway, Co. Roscommon and Co. Mayo, Ireland) and by the HSE Research Ethics Committee Midlands Area and Corporate (Co. Wicklow, Dun Laoghaire, Dublin South East, Co. Laois, Co. Offaly, Co. Longford, Co. Westmeath, Co. Louth and Co. Meath, Ireland) (RRECB1023CC; on 17 January 2024). Adverse events will be recorded and reported to the UCD and relevant ethics committees.

Written informed consent will be obtained at the baseline assessment in the participant’s home prior to any study related procedures being undertaken. The consent form is available at the UCD Research Data Zenodo Community repository (Appendix C) ^45^.

### Declaration of Generative AI and AI-assisted technologies in the writing process

The authors declare that they have not used Generative AI and AI-assisted technologies in the writing process.

## Data Availability

No data are associated with this article. Zenodo: Protein Supplement and Exercise Training for the Treatment of Sarcopenia Risk in Older Adults (POWER), https://doi.org/10.5281/zenodo.14856935 ^45^. This project contains the following extended data: SPIRIT checklist Nutritional content of the ONS Participant consent form Study design and participant flow figure Data are available under the terms of the Creative Commons Attribution 4.0 International license (CC-BY 4.0). UCD Research Data Zenodo Community: This protocol study will be reported following the Standard Protocol Items: Recommendations for Interventional Trials (SPIRIT) with a summary checklist reported (Appendix A) for ‘Oral nutritional supplement combined with an online resistance training programme to improve physical function and nutritional status in older adults receiving home care and at risk of sarcopenia: protocol for the randomised controlled POWER trial’ https://doi.org/10.5281/zenodo.14856935 ^45^. Data are available under the terms of the Creative Commons Attribution 4.0 International license (CC-BY 4.0).
