# Supplementary material for: Oral nutritional supplement combined with an online resistance training programme to improve physical function and nutritional status in older adults receiving home care and at risk of sarcopenia:  protocol for the randomised controlled POWER trial
Source: HRB Open Res. 2026 Jan 20;8:41. Originally published 2025 Mar 17. [Version 3] doi: 10.12688/hrbopenres.14086.3 (PMC12744209; doi:10.12688/hrbopenres.14086.3)
Supplement: Peer review history

# Reviewer report: Reviewer response for version 3

**DOI:** 10.21956/hrbopenres.15781.r52999

**Published:** 2026-01-21

**Authors:** Anne Griffin

**Affiliations:** 1 University of Limerick, Limerick, Ireland

No further comments to make.

Is the study design appropriate for the research question?

Yes

Is the rationale for, and objectives of, the study clearly described?

Yes

Are sufficient details of the methods provided to allow replication by others?

Yes

Are the datasets clearly presented in a useable and accessible format?

Not applicable

Reviewer Expertise:

My research interests focus on the development and optimisation of nutrition care pathways for older adults, aiming to enhance their health outcomes and quality of life. I am particularly interested in addressing the challenges of food security within the realm of public health nutrition, exploring innovative strategies to ensure that all individuals have access to adequate, nutritious food. By examining the intersection of nutrition care and food security, I aim to contribute to the creation of comprehensive, equitable public health policies and practices that support the well-being of older populations.

I confirm that I have read this submission and believe that I have an appropriate level of expertise to confirm that it is of an acceptable scientific standard.

# Reviewer report: Reviewer response for version 2

**DOI:** 10.21956/hrbopenres.15701.r51648

**Published:** 2025-12-27

**Authors:** Miriam E Clegg

**Affiliations:** 1 Food and Nutritional Sciences, University College Cork School of Food and Nutritional Sciences (Ringgold ID: 374114), Cork, County Cork, Ireland

This is an interesting article that presents a protocol for a randomized controlled trial using resistance exercises and dietary protein on physical function (Timed Up and Go) and nutritional status (Mini-Nutritional Assessment-Full Form) as well as health outcomes in older adults in the community who are receiving homecare. The authors provide clear rationale highlighting that this type of intervention has been successful in the hospitalised older adult, but it is not clear if it would be successful in community dwelling older adults who are receiving homecare. In this situation providing an exercise intervention that meets the needs of the older adults can be difficult which makes this online intervention novel. The article is well written and makes good use of current evidence.

 The abstract background could be improved by including a short rationale for the work if there is space.

 Will any data be collected on qualitative aspects of the intervention outside of the questionnaire (e.g. focus group or open-ended questions? It would be interesting to see how much the older adults enjoyed the exercise intervention, how they would see it improved and if technology and lack of in-person support was a barrier.

 Will the participants be given any instructions on continuing the exercise on their own after the intervention? It isn’t very clear what happens between T2 and T3.

 Will the research data be made openly available? There are details on protocol data but I couldn’t see research data – apologies if I missed it.

 My congratulations on the project, I hope that everything goes well and I look forward to seeing the results.

Is the study design appropriate for the research question?

Yes

Is the rationale for, and objectives of, the study clearly described?

Yes

Are sufficient details of the methods provided to allow replication by others?

Yes

Are the datasets clearly presented in a useable and accessible format?

Not applicable

Reviewer Expertise:

Nutrition and healthy ageing

I confirm that I have read this submission and believe that I have an appropriate level of expertise to confirm that it is of an acceptable scientific standard.

# Response

**Published:** 2026-01-08

**Authors:** Catherine Fallon

**Affiliations:** University College Dublin School of Public Health Physiotherapy and Sports Science, Dublin, Leinster, Ireland

Many thanks for your detailed review of our protocol. We truly appreciate the time and care you put into your feedback. We have addressed all comments and have updated the protocol to reflect your suggestions.

1. The abstract background could be improved by including a short rationale for the work if there is space.

Thank you for this suggestion which was also made by Reviewer 1. We have now added a brief rationale to the abstract on the need for combined nutrition and resistance training interventions in older adults at risk of sarcopenia.

 [Abstract]

 Background: *Risk of sarcopenia and poor nutritional status are observed in older adults receiving home care. Hence, the* aim of the POWER randomised controlled trial is to investigate the effectiveness of a whey protein oral nutritional supplement (ONS) combined with an online resistance training (RT) programme compared to RT alone on physical function, nutritional status and health-related outcomes in community-dwelling older adults receiving supportive home care who are at risk of sarcopenia.

 2. Will any data be collected on qualitative aspects of the intervention outside of the questionnaire (e.g. focus group or open-ended questions? It would be interesting to see how much the older adults enjoyed the exercise intervention, how they would see it improved and if technology and lack of in-person support was a barrier.

 Thank you for raising this point.  Yes, qualitative data will be collected at the post-intervention assessment.  Acceptability of both the ONS and online RT programme will be assessed using bespoke questionnaires developed by the research team. These will include hedonic and other Likert-scale items alongside optional free text comments. The free text comments will be analysed using thematic analysis.  We have now included this in the analysis plan.

*[Under statistical analysis plan]*

*Qualitative data analysis plan*

*The free text comments of the acceptability questionnaires will be used to further explore the acceptability of the ONS and online RT intervention and relevant barriers and facilitators to the intervention. Braun and Clarke’s framework of thematic analysis will be used to identify themes and subthemes. This includes familiarisation with the data, creating initial codes, searching for themes, reviewing potential themes and finally, defining themes and subthemes.*

 3. Will the participants be given any instructions on continuing the exercise on their own after the intervention? It isn’t very clear what happens between T2 and T3.

 Thank you for highlighting this.  Between T2 and T3, participants will not receive the online RT programme. At the T2 assessment, they will be encouraged to continue exercising.  We have now clarified this as follows in the RT programme section:

 [under ***Online resistance training programme*]**

*Following the* *T2* *post-intervention assessment, participants will not receive the online RT programme. At the T2 assessment, they will be encouraged to continue to exercise but no additional supervised sessions or support will be provided between T2 and T3.*

 4. Will the research data be made openly available? There are details on protocol data but I couldn’t see research data – apologies if I missed it.Study participants will be informed that their data will be analysed as pseudonymised data to investigate the effects of the intervention on the primary outcomes: functional status as measured by TUG and nutritional status as measured by MNA-FF.  They will also be informed that data on secondary outcomes will be analysed i.e., body composition, dietary intake, gait speed, muscle strength, cognitive function, depression risk, activities of daily living, quality of life and feasibility of intervention implementation. The data will be published in appropriate peer-reviewed publications and presented at suitable research conferences. No participant personal data or data that could potentially identify participants will be published. This is now included under Data Management.

 [Under data management section]

 Personal data will be stored in a password protected university database in compliance with the Data Protection Act 2018 ^78^ . Only the researchers will have access to this information.  Each participant will be assigned a study identification number, which will be used to pseudonymise the data collected. All data will be pseudonymised for analysis, enabling the potential re-linking of individual participant data for data checking if necessary. All data will be anonymised after four years. No personal data will be transferred to Nutricia Ireland Ltd as per the signed legal agreement between UCD and Nutricia Ireland. *Study data will be made available to researchers upon reasonable request.*

 My congratulations on the project, I hope that everything goes well and I look forward to seeing the results.

 We appreciate the reviewer’s positive feedback on our protocol paper.

# Reviewer report: Reviewer response for version 2

**DOI:** 10.21956/hrbopenres.15701.r51370

**Published:** 2025-11-20

**Authors:** Anne Griffin

**Affiliations:** 1 University of Limerick, Limerick, Ireland

Summary of the article:  This article describes the protocol for the POWER randomized controlled trial that will evaluate the effectiveness of a fortified, whey protein ONS and online resistance training exercise class among older adults at risk of sarcopenia. The primary outcomes are physical function, nutritional status and health-related outcomes.  The novelty of this research is that the cohort are community dwelling older adults in receipt of home care (formal or informal).

 Thank you for the opportunity to review this article.  I have suggestions that I hope support your work.

 Background: Inclusion of a statement of rationale for the POWER trial would provide some background context, e.g. relevant to sarcopenia.

 Conclusion: A sentence outlining the significance of any findings from the RCT (why does it matter) could be included.  

 Background: Check grammar as currently unclear if you intend that the ONS product is novel or the approach (ONS and online RT) is novel. I think it is the latter?

 Methods: Eligibility criteria, recruitment and screening: Please provide a clear rationale for excluding older adults with cognitive impairment from your study. Contemporary guidance emphasises the importance of including this population or, where exclusion is necessary, explaining why and how this decision was made.  

 Dietary intake: Please provide references for the technique described.

 Discussion: Well written and the limitations of the trial are well considered.

Is the study design appropriate for the research question?

Yes

Is the rationale for, and objectives of, the study clearly described?

Yes

Are sufficient details of the methods provided to allow replication by others?

Yes

Are the datasets clearly presented in a useable and accessible format?

Not applicable

Reviewer Expertise:

My research interests focus on the development and optimisation of nutrition care pathways for older adults, aiming to enhance their health outcomes and quality of life. I am particularly interested in addressing the challenges of food security within the realm of public health nutrition, exploring innovative strategies to ensure that all individuals have access to adequate, nutritious food. By examining the intersection of nutrition care and food security, I aim to contribute to the creation of comprehensive, equitable public health policies and practices that support the well-being of older populations.

I confirm that I have read this submission and believe that I have an appropriate level of expertise to confirm that it is of an acceptable scientific standard.

# Response

**Published:** 2026-01-08

**Authors:** Catherine Fallon

**Affiliations:** University College Dublin School of Public Health Physiotherapy and Sports Science, Dublin, Leinster, Ireland

Many thanks for taking the time to review our protocol. We appreciate your thoughtful comments and feedback. We have carefully considered all points raised and have amended the protocol accordingly.

1. **Abstract Background: Inclusion of a statement of rationale for the POWER trial would provide some background context, e.g. relevant to sarcopenia.**

Thank you for this suggestion. We have now added a brief rationale to the abstract on the need for combined nutrition and resistance training interventions in older adults at risk of sarcopenia. We have also amended the Methods section of the abstract to keep within the word count.

 [Abstract]

*Background Risk of sarcopenia and poor nutritional status are observed in older adults receiving home care. Hence, the *aim of the POWER randomised controlled trial is to investigate the effectiveness of a whey protein oral nutritional supplement (ONS) combined with an online resistance training (RT) programme compared to RT alone on physical function, nutritional status and health-related outcomes in community-dwelling older adults receiving supportive home care who are at risk of sarcopenia.

 Participants in the ONS + RT group will be provided with a whey protein ONS enriched with leucine and vitamin D to consume twice *daily* for 12 weeks. All participants will be provided with an online RT programme twice *weekly* via Zoom.

1. **Abstract Conclusion: A sentence outlining the significance of any findings from the RCT (why does it matter) could be included.  **

Thank you for this suggestion. We have added a sentence to the conclusion of the abstract outlining the potential significance of the POWER trial for clinical practice.

 Conclusion This study will provide data on the effectiveness of a whey protein ONS enriched with leucine and vitamin D combined with an online RT programme compared to the RT programme alone for older adults at risk of sarcopenia and receiving supportive home care. *If effective, this combined approach could act as an important strategy to support maintenance of physical function and nutritional status in older adults, for whom home-based interventions are likely a more feasible option.*

1. **Background: Check grammar as currently unclear if you intend that the ONS product is novel or the approach (ONS and online RT) is novel. I think it is the latter?**

Thank you for this suggestion.  Nutricia refers to the ONS as a novel ONS as it has only been used in clinical trials to date. We have discussed the phrasing as a team and believe that the novelty relates to the approach in the population studied rather than the ONS per se. As such, we think that it is better to omit the use of the word ‘novel’.

 Background

*Hence*, the aim of this study is to test the effectiveness of a *new *whey protein ONS, enriched with leucine and vitamin D (Fortimel Advanced, Nutricia) combined with an online RT programme versus the RT programme alone on the physical function and nutritional status of older adults receiving supportive home care who are at risk of sarcopenia."

1. **Methods: Eligibility criteria, recruitment and screening: Please provide a clear rationale for excluding older adults with cognitive impairment from your study. Contemporary guidance emphasises the importance of including this population or, where exclusion is necessary, explaining why and how this decision was made.  **

Thank you for highlighting this important point. Our rationale for excluding older adults with an MMSE <24 was twofold: i) the need for older adults to follow instructions independently at home to deliver an effective exercise dose and ii) our concern about potential risk of injury given the online delivery of the resistance training programme. However, we would hope to see future research provide a similar intervention to older adults with cognitive impairment that incorporates additional supports to allow safe and effective exercise delivery. This could include having a trained family member/care assistant with the older person for the exercise sessions.

 [Under Eligibility criteria, recruitment and screening]

*Older adults with moderate to severe cognitive (MMSE <24) impairment will be excluded due to the need for older adults to follow instructions independently at home to deliver an effective exercise dose and our concern about potential risk of injury given the online delivery of the resistance training programme.*

1. **Dietary intake: Please provide references for the technique described.**

Thank you.  We have added references supporting the dietary intake assessment method used.

 [Under Dietary intake]

 A 24-hour dietary recall using a multiple pass technique will be conducted at each home-based assessment to establish the dietary pattern of each participant *(Steinfeldt, Anand and Murayi, 2013).*

1. **Discussion: Well written and the limitations of the trial are well considered.**

We appreciate the reviewer’s positive feedback on the Discussion section.
